# Regridding reconstruction algorithm for real-time tomographic imaging

**DOI:** 10.1107/S0909049512032864

**Published:** 2012-09-01

**Authors:** F. Marone, M. Stampanoni

**Affiliations:** a Swiss Light Source, Paul Scherrer Institut, Villigen, Switzerland; b Institute for Biomedical Engineering, University and ETH Zurich, Zurich, Switzerland

**Keywords:** tomographic reconstruction, fast algorithm, Fourier method, regridding, PSWF

## Abstract

Sub-second temporal-resolution tomographic microscopy is becoming a reality at third-generation synchrotron sources. Efficient data handling and post-processing is, however, difficult when the data rates are close to 10 GB s^−1^. This bottleneck still hinders exploitation of the full potential inherent in the ultrafast acquisition speed. In this paper the fast reconstruction algorithm *gridrec*, highly optimized for conventional CPU technology, is presented. It is shown that *gridrec* is a valuable alternative to standard filtered back-projection routines, despite being based on the Fourier transform method. In fact, the regridding procedure used for resampling the Fourier space from polar to Cartesian coordinates couples excellent performance with negligible accuracy degradation. The stronger dependence of the observed signal-to-noise ratio for *gridrec* reconstructions on the number of angular views makes the presented algorithm even superior to filtered back-projection when the tomographic problem is well sampled. *Gridrec* not only guarantees high-quality results but it provides up to 20-fold performance increase, making real-time monitoring of the sub-second acquisition process a reality.

## Abstract 2

A fast algorithm for tomographic reconstruction based on the Fourier method is presented. On CPU, it provides an up to 20-fold performance increase compared with filtered back-projection routines with negligible accuracy degradation.

## 1. Introduction

At third-generation synchrotron facilities, highly brilliant X-rays coupled with modern detector technology permit routine acquisition of high-resolution tomograms in a few minutes, making high-throughput experiments a reality (Hintermüller *et al.*, 2010 ▶; Marone *et al.*, 2010 ▶; De Carlo *et al.*, 2006 ▶; Rivers *et al.*, 2010 ▶; Chilingaryan *et al.*, 2010 ▶). Recently, the latest detectors based on CMOS technology (Baker, 2010 ▶) have been tremendously pushing the achievable temporal resolution bringing real-time tomography closer. This hardware advance is paving the way to new science and making new experiments possible that until recently were unimaginable, where dynamic processes can for the first time be captured in three dimensions through time (Mokso *et al.*, 2010 ▶; Di Michiel *et al.*, 2005 ▶). For instance, the study of evolving liquid and metallic foams, the investigation of alloys under thermal or mechanical stress, and the imaging of living animals giving insight into physiological phenomena are only a few examples of various challenging applications that will extremely benefit from sub-second temporal-resolution tomographic microscopy.

A full tomographic dataset consists of a series of X-ray projection images acquired with the sample at different orientations around a vertical rotation axis. These images are subsequently combined using tomographic reconstruction algorithms to obtain the three-dimensional structure of the investigated specimen. A high-resolution projection series typically consists of more than a thousand images and the projection size is usually of the order of 2000 × 2000 pixels. Tomographic microscopy featuring both sub-second temporal resolution and micrometer spatial resolution is therefore intrinsically coupled to an extremely high data rate (up to 10 GB s^−1^). As a consequence, to fully exploit the potential provided by sub-second temporal resolution, new solutions for efficient handling and fast post-processing of such a large amount of data are mandatory. Post-processing and tomographic reconstruction of raw datasets should ideally occur on a similar time scale as their acquisition, so that data collection and reconstruction can go in parallel, allowing online quality assessments and data evaluation.

Filtered back-projection (FBP) has been the standard reconstruction method for many years (Kak & Slaney, 2001 ▶). For scan times of the order of tens of minutes to hours and usual projection sizes not exceeding 1024 × 1024 pixels, FBP algorithms running on small CPU (central processing unit) clusters were able to provide full tomographic reconstructions in a time frame similar to that for the data acquisition. With the advent of third-generation synchrotron sources and new detectors, this is no longer the case and new high-performance computing solutions are mandatory.

Recently, emerging GPU (graphics processing unit) technology has attracted a lot of interest and is starting to be successfully exploited, mostly integrated with CPUs to create hybrid architectures, for the acceleration of tomographic reconstructions in different fields making use of standard FBP algorithms (De Witte *et al.*, 2010 ▶; Chalmers, 2011 ▶). A GPU is still, however, a relatively specific hardware component and specialized knowledge for the implementation of software optimized for this novel architecture is necessary, but not always readily available in-house.

In this paper an alternative algorithm to the standard FBP routine, highly optimized for conventional CPU technology, is presented and discussed. This fast reconstruction approach is based on the Fourier transform method (FTM). The critical step of such a method, the regridding of the Fourier space, is performed by convolution of the data in the Fourier domain with the Fourier transform of functions with particular characteristics [one-dimensional (1D) prolate spheroidal wavefunctions], enabling excellent performance without accuracy degradation.

In the following, first the mathematical background is laid out and critical implementation issues are considered. Then the accuracy of the reconstructions delivered by the described algorithm is assessed using both synthetic and real datasets. Finally the performance of this FTM is discussed.

## 2. Fourier transform methods

According to the Fourier slice theorem (Kak & Slaney, 2001 ▶), the Fourier transform of a parallel projection of an object obtained at angle ϕ equals a line of the two-dimensional (2D) Fourier transform of the object taken at the same angle. Making use of this theorem, the 2D Fourier space can be filled with the Fourier transforms of parallel projections of an object taken at different angles. Specifically for X-ray absorption tomography, information on the linear absorption coefficient of the studied object can then be recovered by a 2D inverse Fourier transform of the Fourier space, if this is sufficiently sampled. Hence, such a tomographic reconstruction process consists of a series of 1D Fourier transforms followed by a 2D inverse Fourier transform.

Instead of the 2D inverse Fourier transform, FBP routines exploit the analytic inverse Radon transform. It can be shown (Kak & Slaney, 2001 ▶) that the reconstructed image at a certain point is the summation of all projection samples that pass through that point, after a filter has been applied; or, in other words, the back-projection operation uniformly propagates the measured projection value back into the image along the projection path.

### 2.1. Interpolation

The critical step of FTMs, which prevented until recently their wider application, is the interpolation in the Fourier space from polar to Cartesian grid required for efficient computation of the 2D inverse fast Fourier transform (FFT). In fact, interpolation in the frequency domain is not as straightforward as interpolation in real space. In direct space, an interpolation error is localized to the small region where the pixel of interest is located. This property does not hold, however, for interpolation in the Fourier domain, because each sample in a 2D Fourier space represents certain spatial frequencies and contributes to all grid points in direct space. Therefore, an error produced on a single point in Fourier space affects the appearance of the entire image (after inverse Fourier transform). It has been shown (Choi & Munson, 1998 ▶; O’Sullivan, 1985 ▶) that optimal interpolation using sinc functions is possible. However, owing to its heavy computational burden caused by the infinite extent of the sinc function, this approach soon appeared unviable. Various alternative interpolation techniques (linear, bilinear, splines, *etc.*) have also been considered, but a trade-off between accuracy and speed exists: with reasonable computational efforts the quality of FBP reconstructions has never been achieved.

Owing to the need of using an iterative approach to overcome missing data outside the resolution circle (Miao *et al.*, 2005 ▶), inevitably leading to longer reconstruction times, the pseudo-polar FFT (Averbuch *et al.*, 2008 ▶), an exact FFT algorithm relating the pseudopolar and the Cartesian grid, is also not an option.

As an alternative, the algorithm for tomographic reconstructions presented here, initially introduced by Dowd *et al.* (1999 ▶) and named *gridrec*, makes use of the gridding method for resampling the Fourier space from polar to Cartesian coordinates, offering both computational efficiency and negligible artifacts. The general gridding approach was originally proposed in radio astronomy (Brouw, 1975 ▶) to back-transform irregularly sampled Fourier data and later introduced in computerized tomography by O’Sullivan (1985 ▶). In the gridding technique the data in the Fourier space are mapped onto a Cartesian grid after convolution with the Fourier transform of a certain function *w*(*x*, *y*), whose contribution is removed after the 2D inverse FFT. The idea is to pass a convolution kernel over the data sampled on the polar grid with the convolution output evaluated at the points of the Cartesian grid. The success of the method depends on the rate of decay of the convolution kernel outside the region of interest compared with the values within. For best reconstruction accuracy and minimal aliasing [introduced by the uniform spacing of the Cartesian grid (O’Sullivan, 1985 ▶)], the convolution kernel *w*(*x*, *y*) needs to be well concentrated in the region of interest and its Fourier transform should vanish for spatial frequencies larger than a few grid spacings. The compact support of these functions required for reconstruction accuracy also guarantees the necessary computing performance.

Here we use a separable form for *w*(*x*, *y*) = *w*(*x*)*w*(*y*), with *w*(*x*) chosen from the family of 1D prolate spheroidal wavefunctions (PSWFs) of zeroth order (Slepian & Pollak, 1961 ▶). In fact, it has been shown (Slepian & Pollak, 1961 ▶; Landau & Pollak, 1961 ▶, 1962 ▶; Slepian, 1964 ▶) that these functions best satisfy the requirement for maximal concentration of a time-limited function to a limited bandwidth.

PSWFs cannot be expressed by means of well studied functions and are difficult to calculate exactly. Nonetheless simple accurate approximations exist, enabling the efficient computation and storage of these functions and their Fourier transforms at run time, using known rapidly converging expansions of PSWFs in terms of Legendre polynomials (Van Buren, 1975 ▶; Xiao *et al.*, 2001 ▶). For highest reconstruction accuracy it is, however, important to consider a sufficiently high expansion degree.

To prevent confusion it must be pointed out that interpolation and discrete convolution are equivalent if the basis functions used for interpolation are convolutional, *i.e.* if the basis is constructed by integer shifts of a single function. This is the case in FTMs, where the words interpolation and convolution can be, and actually often are, exchanged.

### 2.2. Mathematical formulation

In the following the equations governing tomographic reconstructions are laid out from a viewpoint of direct FTMs rather than, as usually done, from the perspective of FBP. In particular the relationship between the 2D Fourier transform of the object under study and the acquired data is shown. This section should enable a better understanding of FTMs and of the critical steps inherent in the implementation of tomographic reconstruction algorithms.

**Figure 1:**
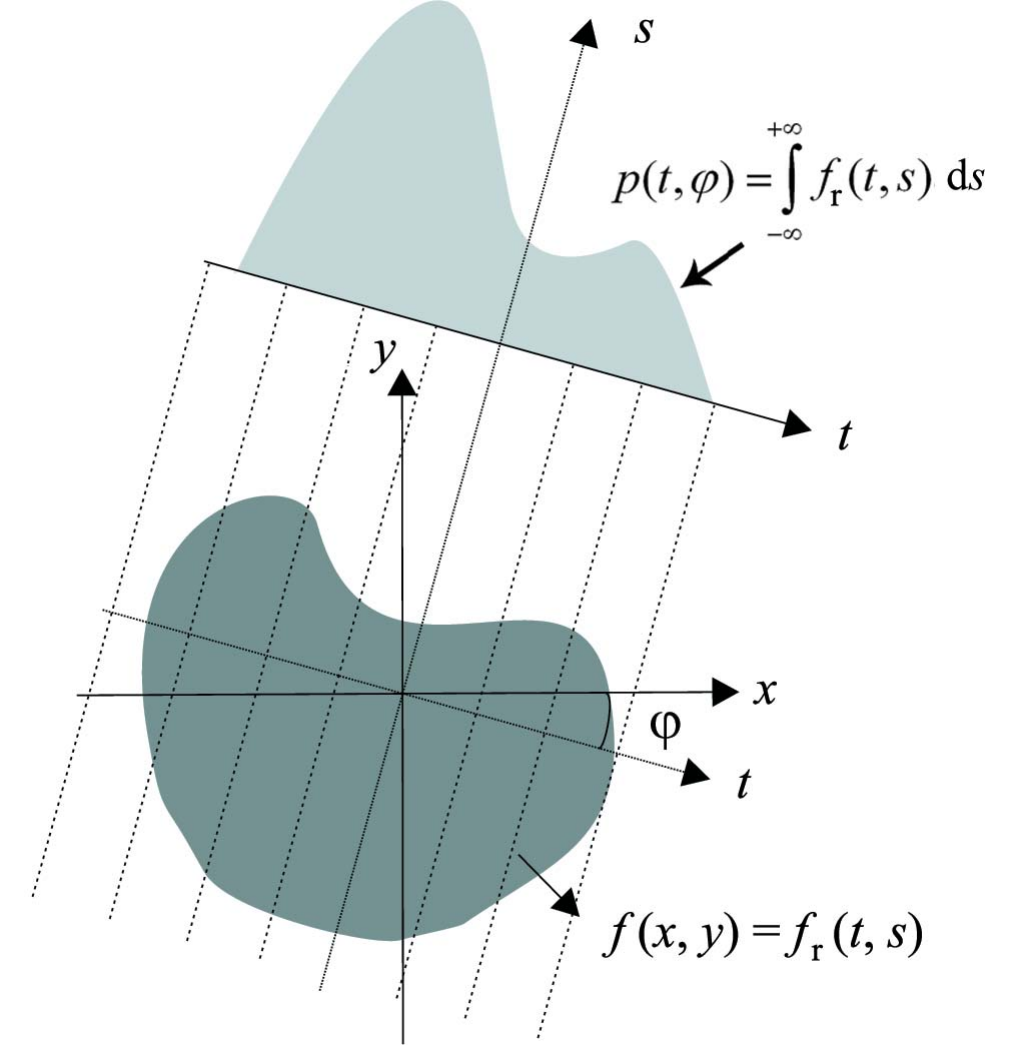
Original and rotated coordinate system used.

We define the original and rotated coordinate system according to the sketch in Fig. 1 ▶. The function *f*(*x*, *y*) and its equivalent *f*_r_(*t*, *s*) in the rotated coordinate space describe the properties of the object, *e.g.* the linear attenuation coefficient, which one wants to reconstruct. *p*(*t*, ϕ), being a parallel projection of *f*(*x*, *y*) taken at angle ϕ, represents the data actually acquired.

According to the Fourier slice theorem (Kak & Slaney, 2001 ▶), the Fourier transform of *p*(*t*, ϕ) equals a line of the 2D Fourier transform of the object taken at angle ϕ. Making use of this theorem, the 2D Fourier space can be filled with the Fourier transforms of parallel projections of an object taken at different angles. Information on the linear attenuation coefficient of the studied object can then be recovered by a 2D inverse Fourier transform of the Fourier space *F*(*u*, *v*), according to
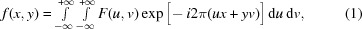
if this is sufficiently sampled.

In practice, *F*(*u*, *v*) is known along radial lines and not on a Cartesian grid as required by (1). To be able to use (1) the Fourier space needs to be mapped from polar coordinates to a Cartesian grid. In gridding algorithms, such as the one presented here, the idea is to pass a convolution kernel *W*(*u*, *v*) over the data sampled on the polar grid, with the convolution output evaluated at the points of the Cartesian grid. The contribution of *W*(*u*, *v*) is then removed after the 2D inverse FFT.

In Cartesian coordinates this convolution step can be expressed as follows,
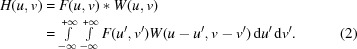
With a transformation to polar coordinates, one obtains
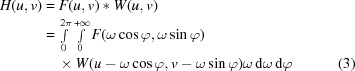
or
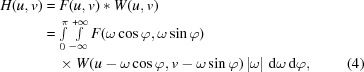
making use of the symmetry property 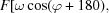 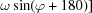 = 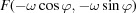, where 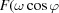, 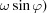 represents the measured data in the Fourier domain.

The multiplication 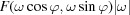 in (4) corresponds to the filtering operation in FBP routines. As is the case for FBP, for FTMs superior reconstructions with smaller noise contamination are also obtained if a smoothing window (*e.g.* Parzen) is additionally used.

The analytical expression (4) calls for integration over all spatial frequencies. In practice the data are discrete and confined in space, therefore band-limited. As a consequence, for the implementation of this method, (4) needs to be discretized. Information about a projection is known in *N* discrete bins and a projection can be represented by 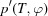 for *T* = 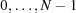. Its Fourier transform is
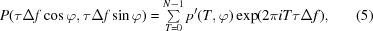
with τ = 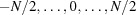 and 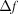 = 1/*N*.

If the bin size is assumed to be 1, a projection in the Fourier domain will only exhibit energy in the frequency interval 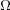 = ±0.5. In addition, during an experiment only a finite number of projections *M* at discrete angles can be acquired. Equation (4) can then be approximated as
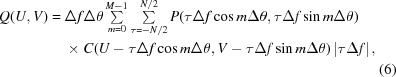
where 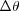 = 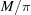.

The unlimited integral over ω in (4) is expressed as a limited sum in the discretized version (6).

### 2.3. Artifacts and solutions

**Figure 2:**
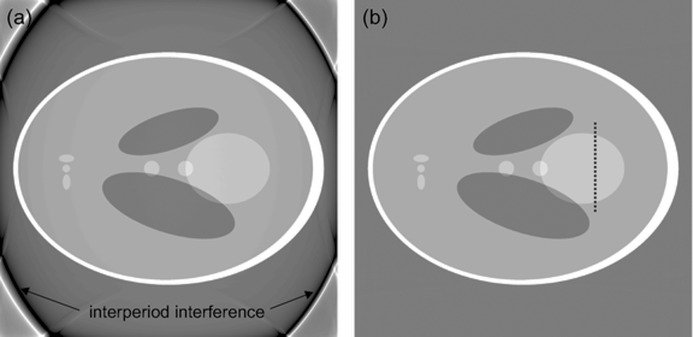
Reconstructed slices of a modified Shepp–Logan phantom (*a*) without zero-padding showing interperiod interference and (*b*) with adequate zero-padding. The dotted line shows where the line profiles in Fig. 4 ▶ are taken. The grey scale has been adjusted to make the features and artifacts more easily discernible. In this way the ellipse contour (pixel value = 1.0) is saturated.

Computer implementation of tomographic reconstruction algorithms, based both on FTMs and FBP routines, can lead to several artifacts adversely affecting the reconstructed images, as a result of the inherent discretization required. In fact, interperiod interference (Fig. 2*a* ▶) and a DC-shift (Fig. 4*a*) can occur (Kak & Slaney, 2001 ▶; Magnusson *et al.*, 1992 ▶) if the nature of the circular convolution and the discretization of the truncated filter kernel are not properly taken into account. Although the recognition of these artifacts and their solution are not new, in implementation of reconstruction algorithms [*e.g.* iradon function in Matlab (MathWorks, Natick, MA, USA)] these issues are nonetheless often neglected. Here we are therefore clearly describing the problem, its origin and appropriate approaches for a clean implementation.

#### 2.3.1. Interperiod interference

By taking into account the discrete, finite and band-limited characteristics of the problem, and therefore moving from an infinite integral in (4) to a finite sum in (6), an aperiodic convolution is converted into a circular convolution, typical for a discrete-time Fourier transform. If the nature of the circular convolution is not properly taken into account, in particular the fact that one of the two functions is assumed to be periodic, some of the convolution terms ‘wrap around’ into the reconstructed image, strongly contaminating the image content. In addition to the clearly visible features at the borders of the reconstruction (Fig. 2*a* ▶), a general cupping with a positive gradient towards the center is also overlaying the image (Marone *et al.*, 2010 ▶), completely compromising the quantitative character of the technique and making the data analysis (*e.g.* segmentation) less straightforward. These aliasing artifacts can easily be overcome by adequately zero-padding the projections (Fig. 2*b* ▶). The minimum number of added zeroes must equal the number of samples in the original projection minus 1.

#### 2.3.2. Constant offset

The discretization of tomographic reconstruction algorithms as described in §2.2 implies zeroing out all information for the frequency interval corresponding to τ = 0 in equation (6), as opposed to the theory [equation (4)], which instead calls for zeroing only at one specific frequency ω = 0. This increased loss of information is responsible for a constant offset in the obtained grey-level values throughout a reconstructed slice.

This artifact can be overcome following a different implementation of (4), which takes into account the band-limited nature of the projection in an alternative way (Kak & Slaney, 2001 ▶),
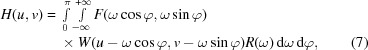
where
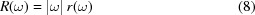
and
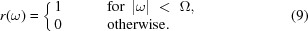
The impulse response 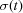 of the filter 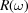 is given by its inverse Fourier transform,
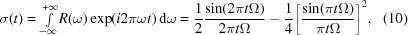
assuming a sampling interval of 1.

**Figure 3:**
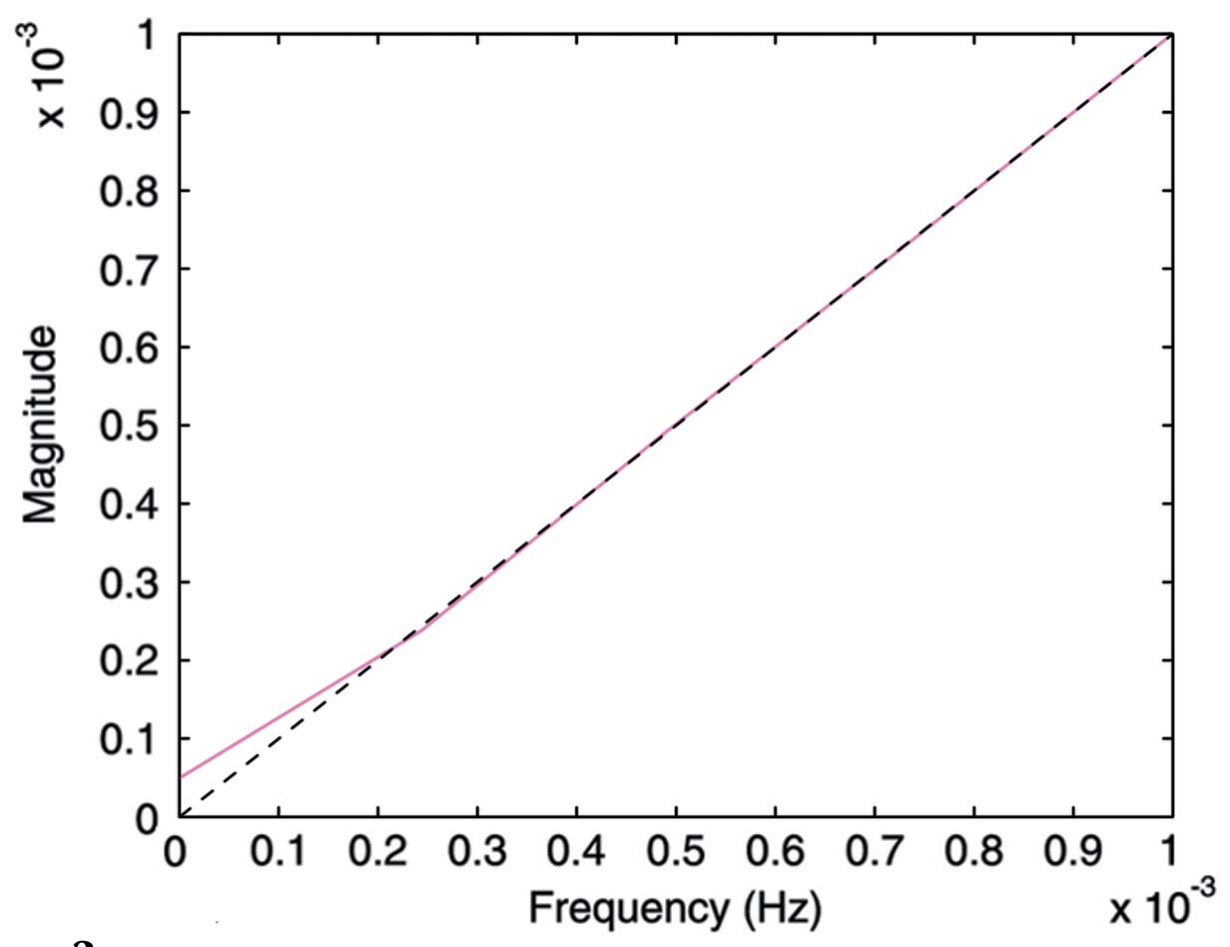
Filter kernel: comparison between the ideal ramp filter |ω| (dashed black) and the discrete Fourier transform of the finite and discrete impulse response of the filter *R*(ω) (pink) in the vicinity of the origin.

**Figure 4:**
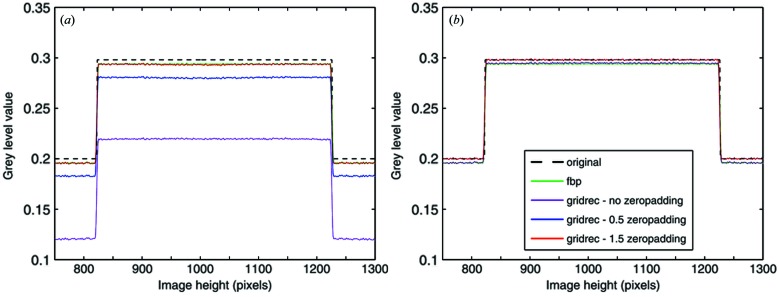
Line profile along the dotted line through the reconstruction of the modified Shepp–Logan phantom in Fig. 2(*b*) ▶ using different algorithms: (*a*) using the approximation in equation (6) resulting in a DC shift, and (*b*) using an appropriate implementation of the discretized truncated filter kernel shown in Fig. 3 ▶.

For a discrete implementation the filter needs to be evaluated only at discrete points,
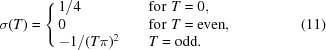
The discrete Fourier transform of 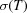 is shown in Fig. 3 ▶ compared with the ideal ramp filter 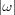. The major difference lies in the DC component. The substitution in (6) of 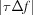 by the discrete Fourier transform of 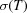 removes the observed constant offset from tomographic reconstructions (Fig. 4 ▶). If (6) is used, a negative offset compared with the original is observed. This offset is dependent on the zero-padding used. In fact, by increasing the zero-padding, one decreases the size of the frequency bin in the Fourier domain, and therefore the loss of information related to zeroing occurring for τ = 0. Although zero-padding can mitigate this artifact, it cannot eliminate it completely, as it is instead possible (Fig. 4*b* ▶) by considering the band-limited nature of the data in this alternative way.

## 3. Accuracy assessment

To assess and highlight different aspects regarding the accuracy of the reconstructions obtained with the presented algorithm, a synthetic and a real dataset have been chosen. The accuracy is investigated using in particular line profiles and histogram plots, since these tools give better insight into the quantitative aspects as opposed to simple visual inspection of 2D reconstructed slices.

### 3.1. Shepp–Logan phantom

**Figure 5:**
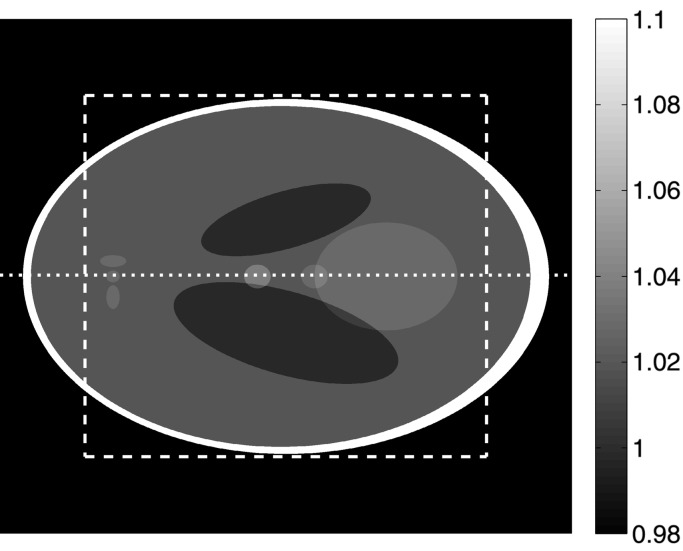
Original Shepp–Logan phantom (Shepp & Logan, 1974 ▶). The grey scale has been adjusted to make the features in the ellipse discernible. In this way the background (pixel value = 0.0) and the ellipse contour (pixel value = 2.0) are saturated. The dotted line shows the position of the line profiles in Figs. 6 ▶ and 7 ▶. The dashed square delimits the area used for the histograms shown in the same figures.

**Figure 6:**
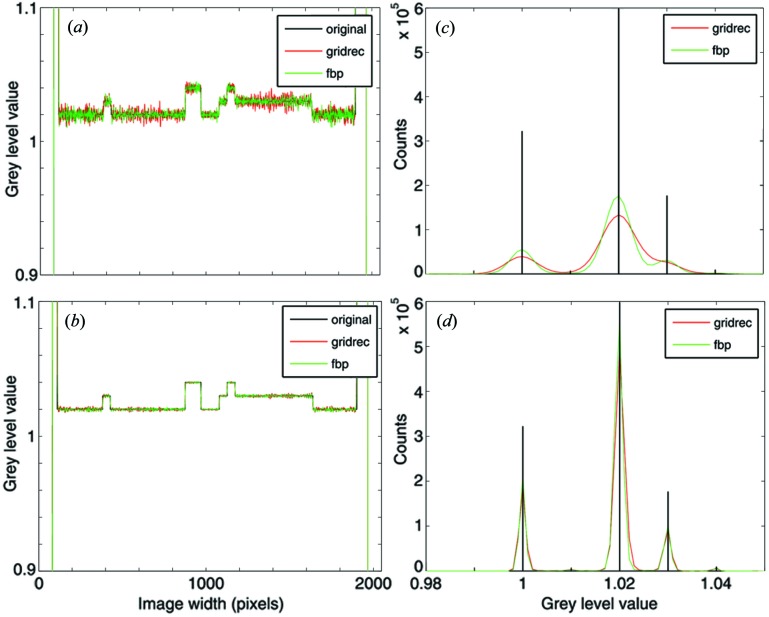
(*a*, *b*) Line profiles along the dotted line in Fig. 5 ▶ and (*c*, *d*) grey-level value histograms for the region delimited by the dashed square in Fig. 5 ▶. Black: original phantom; green and red: reconstructions obtained with FBP and *gridrec*, respectively. For the reconstruction, different filters have been used: Lanczos (*a*, *c*) and Parzen (*b*, *d*). The size of the original phantom used and the reconstructed images is 2048 × 2048 pixels.

The synthetic dataset chosen was the well known Shepp–Logan phantom introduced in 1974 (Shepp & Logan, 1974 ▶) (Fig. 5 ▶) and still in common use today. The used phantoms have been generated with Matlab. Two versions have been taken into account: a high-resolution (2048 × 2048 pixels) and a low-resolution (512 × 512 pixels) case. The corresponding sinograms with 1501 different views over 180° have subsequently been created and reconstructed using the presented algorithm and a standard FBP routine (Huesman *et al.*, 1977 ▶). Since in the used FBP algorithm the filter kernel is not properly implemented (|ω| is used instead of the pink curve in Fig. 3 ▶), in order to be able to compare results, in Figs. 6 ▶ and 7 ▶ an artificial constant offset (0.018) has been added to the FBP reconstructions. Compared with the modified Shepp–Logan phantom also provided by Matlab and previously successfully used for the accuracy assessment of FTMs (Marone *et al.*, 2010 ▶), the standard phantom (Shepp & Logan, 1974 ▶) features more challenging density jumps.

**Figure 7:**
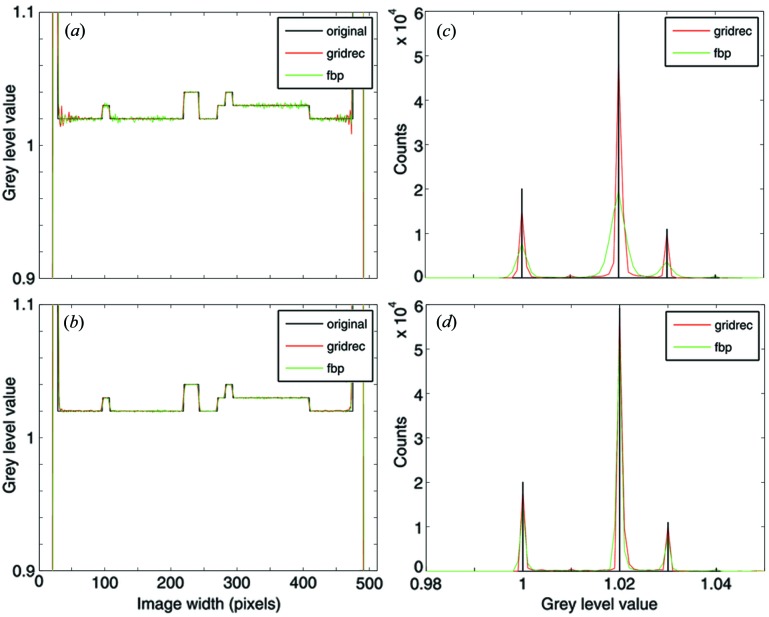
The same as for Fig. 6 ▶, but the size of the original phantom used and the reconstructed images is 512 × 512 pixels.

Line profiles through the reconstructed slices and the corresponding grey-level histograms are shown in Figs. 6 ▶ and 7 ▶ for the high- and low-resolution sinograms, respectively. The line profiles and histograms show a general agreement between the results obtained with FBP and *gridrec*. When the Parzen filter (Huesman *et al.*, 1977 ▶) is used for the reconstruction and therefore the high frequencies are significantly damped, line profiles for the two algorithms are almost not distinguishable [Figs. 6(*b*) ▶ and 7(*b*) ▶]. The comparable reconstruction quality for this case is also confirmed by the grey-level histograms [Figs. 6(*d*) ▶ and 7(*d*) ▶]. On the contrary, if higher frequencies are also considered [*e.g.* by using the Lanczos filter (Duchon, 1979 ▶)], some differences in the noise level are obvious [Figs. 6(*a*) ▶ and 7(*a*)]. This difference is also highlighted by the histograms [Figs. 6(*c*) ▶ and 7(*c*) ▶]. For the high-resolution phantom, *gridrec* reconstructions are visibly noisier than the FBP ones [Figs. 6(*a*, *c*)]; for the low-resolution phantom, the contrary is true [Figs. 7(*a*, *c*) ▶]. These observations hint at the sensitivity of the presented algorithm to the angular sampling of the Fourier space and therefore to the total number of projections acquired for a tomographic scan. To fulfil the sampling theorem, the required number of projections *M* is *M* = *N*π/2, where *N* is the projection width (Kak & Slaney, 2001 ▶). For the high-resolution case, the 1501 views used are not sufficient for satisfying the sampling theorem and the problem is therefore undersampled. For the low-resolution case, 1501 views represent an oversampled problem. Since in FTMs the critical step consists of the resampling of the Fourier space from polar to Cartesian coordinates, the quality of the reconstructions strongly depends on the number of projections used. For FBP routines this dependency is weaker and the reconstruction quality is mainly dominated by the accuracy of the back-projection step. If the Fourier space is strongly undersampled, the interpolation in the Fourier space required by the presented algorithm, in particular for high frequencies where the sampling is sparser, will lack accuracy and the reconstructions will be noisier compared with the results obtained with a back-projection approach. This effect is particularly evident in the Lanczos reconstructions [Figs. 6(*a*) and 6(*c*) ▶], where the high-frequency content is only marginally suppressed. In contrast, if the Fourier space is oversampled, the achieved interpolation accuracy guarantees superior results compared with FBP routines [Figs. 7(*a*) and 7(*c*) ▶]. For a well sampled problem (not shown here), the performance of these two types of algorithms is comparable.

As expected, the chosen spatial sampling of the projections combined with the filter used has an influence on the achieved spatial resolution in the reconstructions. The observed degraded resolution for the 512 × 512 pixel case (Fig. 7 ▶), particularly evident when the Parzen filter is used [rather smooth transitions at density jumps in Fig. 7(*b*) ▶], is however common to both FBP and *gridrec* reconstructions. The limited spatial sampling is also at the origin of ringing/lobe artifacts close to the largest density jump, when the high-frequency content is only slightly suppressed (Fig. 7*a*).

The addition of noise to the synthetic sinograms does not change the overall picture. In particular, if the amount of added noise is comparable with that observed in real data, the trends of the observed reconstruction quality, including the superiority of the presented algorithm for well sampled problems, agree with those for the noise-free case. With increasing noise, the advantage of FTMs in dealing with oversampled problems slowly disappears however.

The resolution degradation inherent in the reconstruction process has also been more rigorously assessed by characterizing the point-spread function for the two approaches. For this purpose a test pattern consisting of 162 points distributed throughout the image in concentric circles has been used. In addition to a high rotation symmetry, all recovered structures, characterized by a bell shape, show a high similarity independent of their position in the image plane, indicating an almost spatially invariant point-spread function. We express resolution in terms of the FWHM. For this purpose we averaged all structures in each reconstruction. Comparison of the FWHM of these mean curves indicates a small resolution degradation when *gridrec* is used. This resolution difference is marginal (about 5%) when Parzen is the chosen filter, and slightly larger (15%) for reconstructions obtained with the Lanczos filter. This observation is independent of the sampling degree of the problem.

### 3.2. Real data

**Figure 8:**
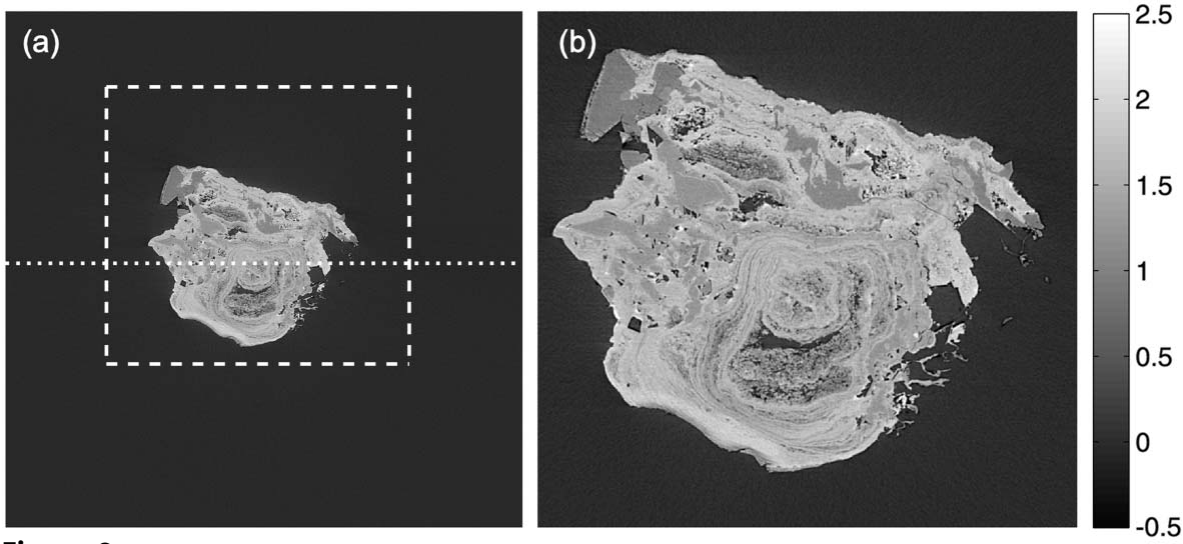
Axial slice through the tomographic reconstruction of a Ca-apatite human kidney stone. (*a*) Overview: the dotted line shows the location of the line profiles in Fig. 9 ▶. The dashed square delimits the area used for the histograms shown in Fig. 9 ▶. (*b*) Magnification of the specimen better illustrating its complexity. [Sample courtesy of A. Pasch, Inselspital Bern, Switzerland. Image acquired at the TOMCAT beamline (Stampanoni *et al.*, 2006 ▶) at the SLS-PSI, Villigen, Switzerland. Pixel size: 1.85 µm.]

In Fig. 8 ▶ an axial slice through a real dataset used for the accuracy assessment of the presented algorithm is shown. The sample is a Ca-apatite human kidney stone measured at the TOMCAT beamline (Stampanoni *et al.*, 2006 ▶) at the Swiss Light Source at the Paul Scherrer Institut. For optimized contrast the used energy was set to 21.5 keV. The specimen was magnified with the 4× objective resulting in a pixel size of 1.85 µm. Over 180°, 1501 equiangularly spaced projections were acquired. Since each projection consists of 2048 × 2048 pixels, this problem is rather undersampled. The used sample is complex, showing both intricate structural features and the presence of different minerals.

**Figure 9:**
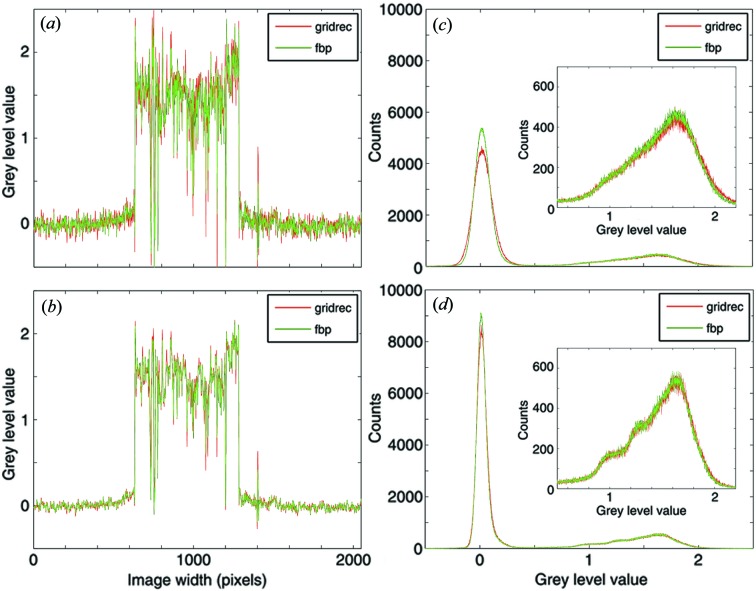
(*a*, *b*) Line profiles along the dotted line in Fig. 8 ▶ and (*c*, *d*) grey-level value histograms for the region delimited by the dashed square in Fig. 8 ▶. Green and red: reconstructions obtained with FBP and *gridrec*, respectively. For the reconstruction, different filters have been used: Lanczos (*a*, *c*) and Parzen (*b*, *d*). The insets in panels (*c*, *d*) represent the magnification of the peak containing the specimen information.

The line profiles [Figs. 9(*a*) and 9(*b*) ▶] through the reconstructions obtained with *gridrec* and FBP show a remarkable agreement. Despite the complex sample structure, a one-to-one correspondence of the wiggles in the line profiles can be observed. For the reconstructions obtained with the Lanczos filter (Fig. 9*a* ▶) where the high-frequency content is only partially suppressed, slightly higher noise is observed in the *gridrec* results, as was the case for the synthetic dataset. If the Parzen filter is used and the high frequencies are therefore more damped, the noise level resulting from the two considered algorithms is more comparable.

These observations are confirmed by grey-level histograms [Figs. 9(*c*) and 9(*d*) ▶], which consist of two major peaks. The sharper peak around zero corresponds to the background, the broader peak on the right to the sample. Although, in the background, *gridrec* reconstructions are slightly noisier than FBP results, accuracy differences in the sample region are minor [Figs. 9(*c*) and 9(*d*) ▶, insets], and, when the Parzen filter is used [Fig. 9(*d*) ▶, inset], basically non-existent, confirming the high reconstruction quality guaranteed by the presented FTM.

Despite the filter kernel in the used FBP algorithm not being properly implemented, a grey-level offset between the reconstructions obtained with the two different methods is hardly visible when real data are used. After careful investigation of the magnified background peak, an offset in the grey level of approximately 1.5 × 10^−6^ can be detected. A small shift between the two histogram curves is also visible in the inset in Fig. 9(*c*) ▶.

A degradation of the spatial resolution when *gridrec* is used is not readily visible.

## 4. Algorithm performance

The main advantage of FTMs over FBP routines lies in the possibility of using the FFT to perform the inverse 2D Fourier transform in a number of steps in the order of *N* ^2^log*N* for an *N* × *N* array, as opposed to *n*_angle_ × *N* ^2^ for standard FBP algorithms, resulting in a significant increase in the reconstruction speed.

For the performance comparison discussed here, a single 2048 × 2048 pixel slice has been reconstructed from a 1501 × 2048 pixel sinogram on a machine equipped with 12 Intel Xeon processors clocked at 2.67 GHz (using though only one single core) and 36 GB RAM. Table 1 ▶ lists the time required for slice reconstructions using different algorithms and amounts of zero-padding (ZP = 0.5 means an extension of each sinogram side by half of the original field of view).

For all reconstructions shown in this work, ZP = 1.5 has been chosen to match the zero-padding inherent in the used FBP routine (Huesman *et al.*, 1977 ▶). In such a case, *gridrec* provides high-quality reconstructions in about one-sixth of the time required by FBP. Moderate zero-padding (ZP = 0.5) is, however, theoretically sufficient to avoid interperiod interference (Kak & Slaney, 2001 ▶). Marone *et al.* (2010 ▶) showed in fact that *gridrec* with ZP = 0.5 also guarantees a comparable quality as FBP. In this case a 20-fold performance improvement is achieved without accuracy degradation.

Also, compared with reconstruction approaches based on FBP routines optimized for hybrid CPU/GPU architectures (Chilingaryan *et al.*, 2010 ▶), *gridrec* performs particularly well. For a reconstruction with moderate padding, *gridrec* is in fact about two times faster (Ferrero, 2011 ▶).

Other reconstruction methods [*e.g.* hierarchical back-projection algorithms (Basu & Bresler, 2001 ▶)] have so far not been considered for this performance comparison.

## 5. Conclusion

At third-generation synchrotron facilities, sub-second temporal-resolution tomographic microscopy is becoming a reality. From the hardware point of view (*e.g.* detectors, photon sources), tremendous progress has been made during the past few years, enabling the acquisition of invaluable new tomographic datasets and therefore promising new science. It is, however, still difficult to fully exploit the potential of this order-of-magnitude increase in temporal resolution, for lack of appropriate solutions for efficient data handling and post-processing, when the generated rates are close to 10 GB s^−1^.

In this paper we demonstrate that the fast reconstruction algorithm *gridrec* is a serious alternative to standard FBP routines. The mathematical details of this FTM are for the first time clearly laid out making this algorithm more accessible to a wider community. Using both synthetic and real datasets we show that this approach guarantees high-quality results. Because it requires interpolation in the 2D Fourier domain, *gridrec* exhibits a stronger dependency on the number of acquired projections compared with FBP. With increasing angular views, the improvement in signal-to-noise ratio for *gridrec* reconstructions is larger than for the case of FBP.

*Gridrec* not only guarantees high-quality results but also provides up to a 20-fold performance increase on standard CPU clusters. Without the need for more specialized technology such as the emerging GPU architecture, ultrafast reconstruction of single tomographic slices is within reach, making real-time monitoring of the sub-second acquisition process a reality. If raw data are readily rearranged into sinogram format during camera read-out, with a moderate size (up to a hundred nodes) CPU cluster high-resolution full tomographic datasets can be reconstructed using *gridrec* in a few seconds, making FTMs interesting for several applications (*e.g.* medical imaging, homeland security), where real-time visualization of the results would be extremely beneficial.

## Figures and Tables

**Table 1:** Algorithm performance

Reconstruction algorithm	Time (s)
*Gridrec*, ZP = 0.5	0.9
*Gridrec*, ZP = 1.5	2.9
FBP, ZP = 1.5	16.7
